# Effects of variations in atmospheric temperature and humidity on the estimation of exclusive breastfeeding status using the deuterium oxide dose-to-mother technique

**DOI:** 10.3389/fped.2023.1188811

**Published:** 2023-11-15

**Authors:** Jeswin Baby, Pernille Kaestel, Tom Preston, Stephen B. Duffull, Zheng Liu, Aly Diana, Lisa Houghton, Anura V. Kurpad, Tinku Thomas

**Affiliations:** ^1^ Division of Epidemiology and Biostatistics, St. John’s Research Institute, Bangalore, India; ^2^ Department of Statistical Sciences, Kannur University, Kannur, India; ^3^ Department of Nuclear Sciences and Applications, International Atomic Energy Agency, Vienna, Austria; ^4^ Scottish Universities Environmental Research Centre, University of Glasgow, Glasgow, Scotland; ^5^ School of Pharmacy, University of Otago, Dunedin, New Zealand; ^6^ Certara, Princeton, NJ, United States; ^7^ School of Medicine and Public Health, Hunter Medical Research Institute, University of Newcastle, Kookaburra Circuit, Newcastle, NSW, Australia; ^8^ Department of Public Health, Faculty of Medicine, Universitas Padjadjaran, Bandung, Indonesia; ^9^ Department of Human Nutrition, University of Otago, Dunedin, New Zealand; ^10^ Department of Physiology, St John’s Medical College, Bangalore, India; ^11^ Department of Biostatistics, St John’s Medical College, Bangalore, India

**Keywords:** deuterium dose to mother technique, exclusive breastfeeding, non-milk oral intake, temperature, humidity

## Abstract

**Background:**

The deuterium dose-to-mother (DTM) method measures the human milk intake of breastfed children. Recently, the use of this method has been expanded to classify babies objectively as exclusively breast fed (EBF) or not (non-EBF) based on quantification of non-milk oral water intake (NMOI). However, the calculation of NMOI estimates involves atmospheric temperature and humidity.

**Objective:**

To evaluate the effects of atmospheric temperature and humidity on NMOI calculation and the classification of exclusive breastfeeding.

**Methods:**

The effect of indoor temperature and relative humidity on NMOI and the estimated prevalence of non-EBF were examined in two existing data sets of DTM in children by varying temperature in the range of 15 to 35°C and relative humidity in the range of 20 to 80% representing the maximum span of indoor conditions expected. Population-level estimates of NMOI distributions were derived using the rstan package for R v2.21.2.

**Results:**

The NMOI decreased at a rate of −1.15 g/day per °C increase and at a rate of −1.01 g/day per percent increase in relative humidity; this was due to variations in non-oral water intake from the atmosphere, a component of the calculation of NMOI, which is dependent on temperature and humidity. For the various locations considered, the mean calculated NMOI varied between 24.6 and 53.3 g/day using the same input data. In the mixed-fed sample of babies, the prevalence of non-EBF based on the earlier defined NMOI cut-off of 86.6 g/day was reduced by 19% when relative humidity was increased by 60%.

**Conclusions:**

Atmospheric conditions are essential factors in the computation of NMOI, used in the objective classification of babies as exclusively breast fed or not, and should be considered when the DTM method is used to classify exclusive breastfeeding.

## 1. Introduction

Suboptimal breastfeeding in the first two years of life is a major contributor to faltering growth and an increased risk of morbidity and mortality (1, 2). The World Health Organization recommends breastfeeding initiation within one hour of birth, followed by exclusive breastfeeding (EBF) up to six months of age. Furthermore, it is recommended that breastfeeding should be continued until 24 months of age, along with appropriate complementary foods after the first six months (3). However, breastfeeding compliance is subjectively and variably self-reported by mothers. Breast milk intake can be objectively measured using the deuterium oxide dose-to-mother (DTM) dilution technique, which measures breast milk transfer from the mother to the baby with reasonable precision (4). While quantifying breast milk intake, the utility of the DTM technique has expanded to include other aspects of breastfeeding practices. For example, because breast milk is considered a complete food for young children (5), this method has been used to compute the requirement for micronutrient intake for zero to six-month-old children. Another recent use of DTM method has been to evaluate whether children are EBF; this is because the “non-milk oral water intake” (NMOI) of the breast fed children is also computed in the DTM method. To define EBF, an NMOI cut-off is required. Several cut-offs for EBF classification have been used, starting with an NMOI of 25 g/day (6). More recently, a cut-off value of 86.6 g/day was proposed (7).

However, assumptions were made in calculating NMOI using the DTM technique (4). The first is a water intake route called non-oral water intake, which occurs via respiration and transcutaneous influx. Water loss may also be caused by respiratory and transcutaneous water efflux (4). Constant values were assumed for these water transactions. Other than the constants associated with non-oral water influx and efflux, the assumed constants were: (1) water influx from the atmosphere was considered as 6.3% of total water intake; (2) a correction for the proportion of deuterium in water loss by breath and transcutaneous water vapor loss, that is subject to isotopic fractionation factor of 0.9919; (3) breast milk water content of 87.1%; and (4) water generated from the oxidation of breast milk as 8.88% (4). Variations in atmospheric temperature and humidity in the surrounding environment can affect water influx, and therefore the estimated NMOI. However, the calculation of breast milk intake does not depend on temperature and humidity.

To the best of our knowledge, there have been no systematic evaluations of the influences of atmospheric temperature and humidity on the constants used in the calculation of NMOI. Therefore, in this study we computationally examined the effect of varying atmospheric temperature and relative humidity on calculating NMOI using the DTM method, which in turn is used in the objective classification of babies as exclusively breast fed or not.

## 2. Methods

**Figure 1:**
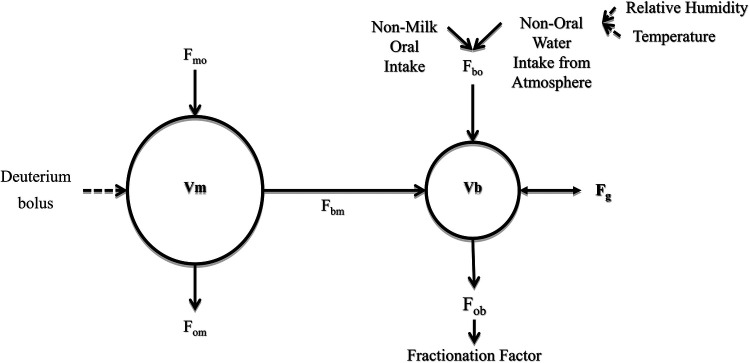
The two-compartment model of water flow between mother-baby pair. F, flow; m, mother; b, baby; o, outside; V, volume of TBW; Vm, mother's total body water volume; Vb, baby's total body water volume; F_mo_, from outside to mother; F_bo_, from outside to baby (non-breast fluid intake); F_bm_, from mother to baby (breast milk intake); F_om_, from mother to outside; F_ob_, from baby to outside (adapted from IAEA human health series No.7) (4).

The DTM method of estimating breast milk transfer from mother to baby involves collecting a series of saliva (body water) samples from the mother and baby over two weeks. The DTM method is based on a two-compartment model first described by Coward et al. (8) as shown in Figure 1.

Four parameters are estimated by minimizing the difference between observed and fitted deuterium enrichment values for mother and baby combined. Parameters namely: Deuterium enrichment in the mother's body water at time zero [Em(0), mg/kg], the transfer of water from the mother to the baby via breast milk (F_bm_, kg/day); the fractional water turnover in the mother (kmm, kg/day) and the total water loss in the baby (F_bb_, kg/day) are estimated. Breast milk intake and Non-Milk Oral Intake estimates are further estimated using these parameters and few assumptions.

### 2.1. Non-milk oral intake

The component of interest in this study was the NMOI (kg/day), which can be estimated from Total water output, Water for growth, Intake of water from breast milk and Non-oral water intake (4).

(1) $$\begin{array}{rl} \mathrm{NMOI}=\; & \mathrm{Total}\;\mathrm{water}\;\mathrm{output}+\mathrm{Water}\;\mathrm{for}\;\mathrm{growth}\\ & \text{–}\;\mathrm{Intake}\;\mathrm{of}\;\mathrm{water}\;\mathrm{from}\;\mathrm{breast}\;\mathrm{milk}\\ & \text{–}\;\mathrm{Non}\text{-}\mathrm{oral}\;\mathrm{water}\;\mathrm{intake} \end{array}$$

### 2.2. Total water output ($F_{\mathrm{ob}}$)

$F_{\mathrm{ob}}$ (kg/day) was estimated from water loss in the baby adjusted by a correction factor for isotopic fractionation (FRAC) (4).

(2) $$F_{\mathrm{ob}}=\frac{F_{\mathrm{bb}}}{\mathrm{FRAC}}$$

FRAC affects water lost from the baby's body as water vapor in the breath and transcutaneous loss. The rate of transcutaneous water efflux (*r*_cE_ g/day) was calculated from the rate of efflux (*r*_bE_** **=** **0.105 g/min/m^2^) and body surface area (BSA) (m^2^).

$$r_{\mathrm{cE}}=0.105\times \mathrm{BSA}\times 60\times 24$$

The BSA (m^2^) of the child is given by $\mathrm{BSA}=0.024265W^{0.5378}H^{0.3964}$ where W(g) and H(cm) are the weight and length of the baby, respectively (9, 10).

The rate of respiratory water efflux (g/day) was calculated as the product of inspired air volume (IAV, L/min) and absolute humidity ($H_{\mathrm{Aexp}},\;\mathrm{mg}/L$) of air expired by the child,

$$r_{\mathrm{bE}}=\frac{\mathrm{IAV}\times H_{\mathrm{Aexp}}\times 60\times 24}{1000}$$

Absolute humidity of expired air by the baby is

$$\;H_{\mathrm{Aexp}}(\mathrm{mg}/L)=216.5\frac{H_{\mathrm{Rexp}}}{273.15+\mathrm{Texp}}$$

Where the relative humidity of expired air (*H*_Rexp_, %), is 94.6% at temperature of expired air $T_{\exp}=35.6^{\circ }C$ and is invariant of atmospheric temperature and humidity (8). The IAV is the product of the resting inspiration of air by the baby (RIA, L/kg/min) and the baby's weight (*W*, kg) (8).

IAV=RIA×W

The RIA was assumed to be 0.25 L/kg/min.

Unfractionated water is equivalent to water loss in babies ($F_{\mathrm{bb}}$). The total water efflux (*r*_tE_, g/day) (8) is given as,

$$r_{\mathrm{tE}}=r_{\mathrm{bE}}+r_{\mathrm{cE}}+(F_{\mathrm{bb}}\times 1000)$$

The fractionation correction is given as

$$\mathrm{FRAC}=\frac{r_{\mathrm{bE}}}{r_{\mathrm{tE}}}F_{b}+\frac{r_{\mathrm{cE}}}{r_{\mathrm{tE}}}F_{c}+\frac{\left(F_{\mathrm{bb}}\times 1000\right)}{r_{\mathrm{tE}}}$$

where the fractionation factors for breath and cutaneous water at body temperature, $F_{b}$ and $F_{c}$, are considered to be 0.946 (4).

### 2.3. Water for growth ($F_{g}$)

$F_{g}$ (kg/day) was estimated from the change in the baby's total body water (TBW, kg), calculated from the baby's initial and final weights, ie: weight at the beginning and end of two-week saliva sampling. TBW of the baby at each sampling time was estimated using Wells' formula (4, 10) $\mathrm{TBW}(t)=0.84\times W(t{)}^{0.82}$.

(3) $$\;F_{g}=\frac{\mathrm{TBW}(T_{N})-\;\mathrm{TBW}(T_{1})}{T_{N}-T_{1}}$$

### 2.4. Intake of water from breast milk ($F_{m}$)

$F_{m}$ (kg/day) was obtained by adding the water content of breast milk to the water produced by the oxidation of nutrients in breast milk.

(4) $$F_{m}=F_{\mathrm{bm}}+(\mathrm{WFO}\times M)$$

where the kinetic variable $F_{\mathrm{bm}}$ (kg/day) provides an estimate of water input from the mother to the baby, WFO is the proportion of water from the oxidation of nutrients in the breast milk, and M is the baby's breast milk intake (kg/day). The calculation of breast milk intake using the DTM method was independent of assumptions regarding atmospheric temperature or humidity.

### 2.5. Non-oral water intake ($F_{a}$)

The non-oral water intake (*F*_a_, kg/day) of a baby is the atmospheric water absorbed through the skin of the baby and the respiratory water influx through the lungs; this was estimated as the product of the proportion of non-oral water intake from the atmosphere (NOWIA) and the total water intake of the baby (4).

(5) $$F_{a}=\;\mathrm{NOWIA}\;(F_{\mathrm{ob}}+F_{g})$$

The total water influx was estimated as the sum of water from the baby to the outside ($F_{\mathrm{ob}}$, kg/day) and $F_{g}$. Respiratory water intake (*r*_bl_- g/day) is calculated as the product of IAV (IAV- L/min) and absolute atmospheric humidity (mg/L) (9). $r_{\mathrm{bl}}=\frac{\mathrm{IAV}\times H_{A}}{1000}$. The absolute atmospheric humidity (*H*_A_, mg/L) was

$$H_{A}=216.5\frac{H_{R}}{273.15+T}$$

where *H*_R_(%) is the relative atmospheric humidity, and T(°C) is the atmospheric temperature. The transcutaneous water influx (*r*_cl_- g/day) was calculated as $r_{\mathrm{cl}}=0.18(\frac{H_{A}}{\mathrm{ASAT}})\mathrm{BSA}$. The formula uses a value for transcutaneous absorption of 0.18 g/m^2^, *H*_A_-absolute humidity (mg/L) *ASAT*- the atmospheric saturation (mg/L) and BSA (m^2^) -the BSA of the baby. Therefore, the influx correction was

$$\mathrm{NOWIA}=\frac{r_{\mathrm{bl}}+r_{\mathrm{cl}}}{(F_{\mathrm{ob}}+F_{g})\times 1000}$$

Thus, atmospheric temperature (°C) and relative humidity (%) influenced the calculation of the NMOI in the DTM method (Figure 1). These calculations demonstrate the quantitative impact of atmospheric temperature and relative humidity on the classification of EBF using the NMOI.

### 2.6. Variations in atmospheric conditions

The temperature and relative humidity were considered to vary in a restricted range, reflecting indoor conditions (11). To demonstrate the effect of these variations, we considered real isotopic enrichment curves of nine babies (body weight: minimum 5.5 kg to maximum 7.8 kg) selected using simple random sampling (details in Supplementary Table S1) from a well-defined sample of children (*n* = 113, body weight 4.0 kg to 7.8 kg) who were observed and verified as EBF (7). The NMOI was computed based on the assumptions used in the International Atomic Energy Agency (IAEA) calculation template (NOWIA = 0.063 and FRAC = 0.9919) (4) and for these children, it ranged from −16 g/day to 124 g/day. In every child, the NMOI calculation can change with respect to atmospheric temperature and relative humidity. The rate of change in NMOI and NOWIA for varying conditions were computed using regression. The variation in NMOI based on simultaneous variation in temperature and relative humidity was also demonstrated in one randomly sampled baby of age 4 months weighing 7 kg from the study sample of 113.

To explore the influence of typical geographical variations in assumed temperature and relative humidity on the calculated NMOI, the entire sample (*n* = 113) of EBF babies in the Indonesian study was used (7, 11, 12). The babies' characteristics are shown in Supplementary Table S2. The values of NMOI that correspond to the 90th percentile of the newly estimated distributions are also presented and compared with the published value of 86.6 g/day to classify into EBF or not (EBF < 86.6 g/day of NMOI) (7). Impact of seasonal variation in temperature and humidity on the estimates in one single location (Yavatmal, India) was also examined. In addition, the variation in NOWIA and NMOI in a single baby (same baby as chosen earlier) at different locations was explored.

In a separate sample of 221 Indonesian partially BF or EBF babies (5) aged two months (1.4 to 2.5 months), the NOWIA and the percentage of babies who were non-EBF (NMOI > 86.6 g/day) were calculated using standard IAEA assumptions (4) and by varying relative humidity from 20 to 80% and temperature from 15 to 35°C. A description of the sample is provided in Supplementary Table S3.

Population-level estimates of the NMOI distributions were derived using the rstan package v2.21.2. R software v4.0.2 was used for data processing and visualization.

## 3. Results

### 3.1. Variation in NMOI in EBF babies

In dataset 1 (nine babies) (Supplementary Table S1), NOWIA increased 1.3 g/day (Supplementary Figure S1A) per °C increase in atmospheric temperature. With increasing relative humidity, NOWIA increased at a rate of 1.15 g/day (Supplementary Figure S1B) per percent relative humidity based on the regression of NOWIA against temperature and relative humidity.

**Figure 2:**
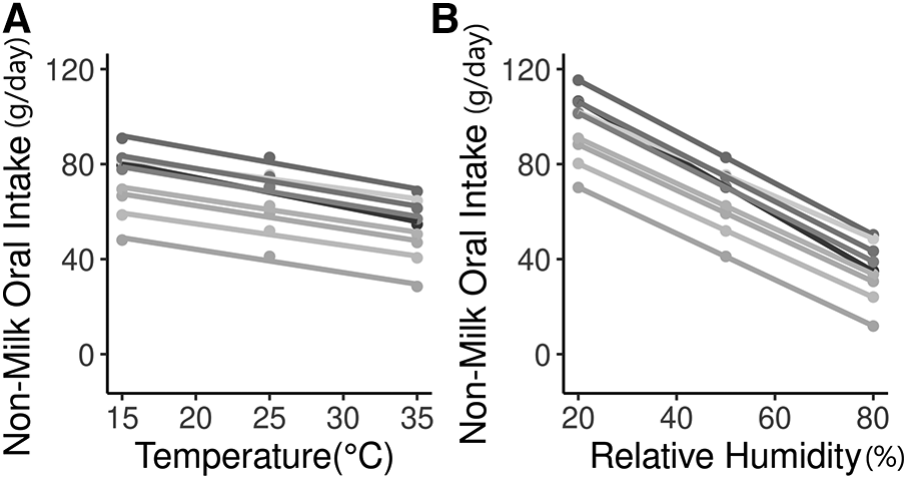
Variation in non-milk oral intake (NMOI, g/day) with temperature (°C) and relative humidity (%). (**A**) varying temperature at constant relative humidity (50%) (**B**) varying relative humidity at constant temperature (25°C). Data from nine randomly selected babies of different weights and body surface areas from the publicly available Indonesian EBF dataset were used.

**Figure 3:**
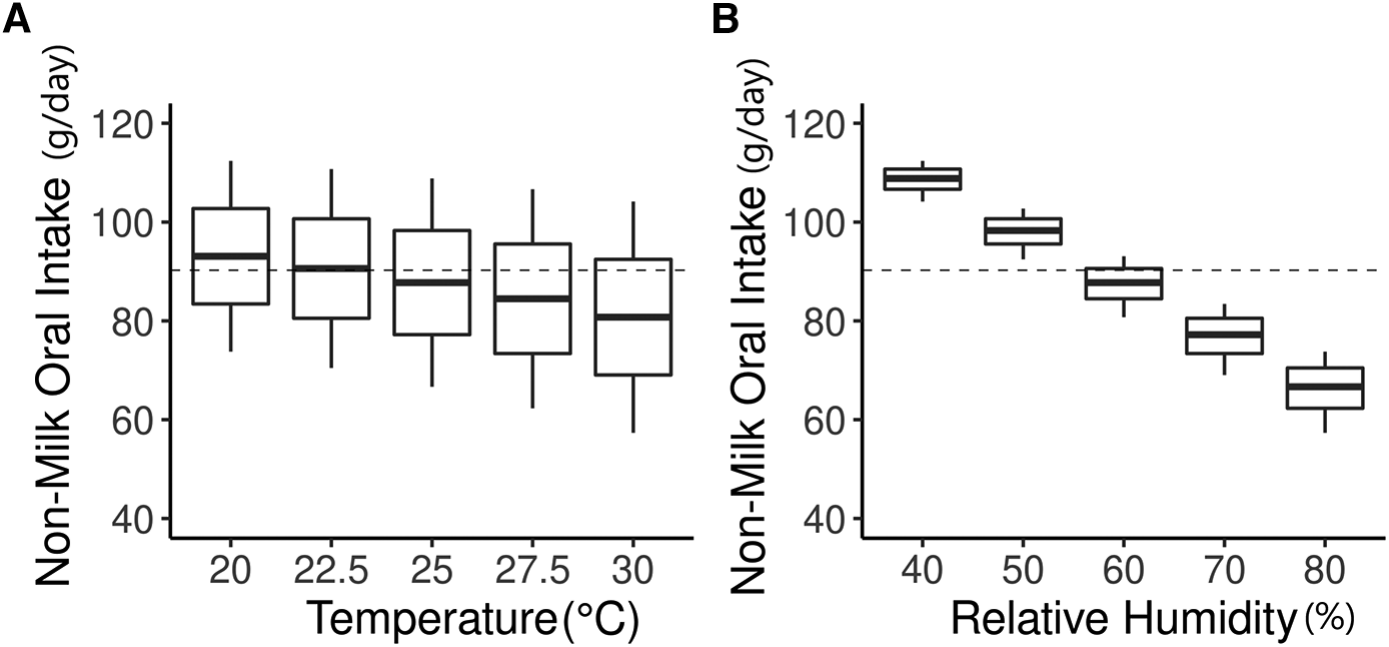
Simulated distribution of non-milk oral intake (NMOI g/day) of a single child. (**A**) varying temperature (°C) levels and (**B**) varying relative humidity (%) levels in a single child with estimated NMOI of 90.2 g/day in original data.

Consequently, the NMOI decreased by −1.2 g/day per °C increase (Figure 2A, relative humidity remained constant at 50%) and by −1.0 g/day per percent increase in relative humidity (Figure 2B, the temperature was kept constant at 25°C). Figure 3 shows the computed distribution of NMOI with variations in temperature (Figure 3A) and relative humidity (Figure 3B) in a single child. The variation in NMOI was greater due to atmospheric relative humidity than to temperature. For a potential range in indoor atmospheric relative humidity between 40 and 80%, the computed NMOI differed by 35.0 g/day (Figure 3B). In addition, the variation in the computed NMOI due to relative humidity increased with increasing temperature, as observed from the box's width in the box-whisker plot (Figure 3B). Similarly, the variation in NMOI due to temperature increased with increasing relative humidity (Figure 3A), demonstrating an interaction between temperature and relative humidity.

Using published average annual indoor temperature and relative humidity across different geographic locations (11, 13), the mean temperature varied from 22.3 to 32.7°C, and the mean relative humidity varied from 42.2 to 75.2% (Table 1); this induced a variation of 61.1 g/day (Bangladesh with highest humidity and relatively high temperature) to 108.7 g/day (United States with lowest humidity and lowest temperature) in computed NMOI in an example baby with 90.3 g/day NMOI computed with the standard assumptions (4). Similarly, using the seasonal variations in indoor temperature and relative humidity in a single location (Yavatmal in India, LAT 20.39° N, LONG 78.13° E) (13), the computed NMOI varied from 63.1 to 122.3 g/day (Supplementary Table S4).

**Table 1:** Variation in intake parameters by mean indoor temperature and relative humidity in different geographical locations (Ref: 11, 13) for one baby as an example.

Location	Indoor temperature (°C)* Mean (SD)	Indoor relative humidity (%)* Mean (SD)	NOWIA (%)	NMOI g/day
Assumed value IAEA (Ref : 4)			0.063	90.3
Blacksburg (US)	22.3 (2.2)	42.2 (14.7)	0.044	108.7
Antananarivo (Madagascar)	23.5 (2.5)	57.3 (12.3)	0.061	92.3
Colombo (Sri Lanka)	29.4 (1.8)	73.3 (8.4)	0.088	66.3
Guatemala City (Guatemala)	22.8 (1.7)	61.6 (7.3)	0.065	88.7
Hong Kong (China)	24.0 (3.0)	63.7 (10.8)	0.068	85.1
Lima (Peru)	23.4 (3.1)	67.6 (8.4)	0.072	81.8
Singapore (Singapore)	27.0 (2.3)	67.6 (9.9)	0.077	76.8
Tuxtla Gutierrez (Mexico)	22.7 (2.4)	68.8 (7.2)	0.072	81.5
Delhi (India)	32.0 (1.4)	64.7 (16.6)	0.082	71.6
Dhaka (Bangladesh)	30.9 (1.4)	75.2 (7.9)	0.093	61.1
Faisalabad (Pakistan)	32.7 (3.5)	53.4 (12.4)	0.069	84.4
Yavatmal (India)	28.9 (4.6)	55.0 (19.6)	0.065	88.2

Water intake from breast milk (cl_mb_rs + rm_rs) remained constant at 815 g/day.

Water used in growth (rg_rs) is a constant value 9.28 g/day, in the calculation.

Isotopic fractionation correction is fixed at 0.9919.

* Values are the mean (SD) of the annual variations in indoor temperature and relative humidity (11, 12) SD, standard deviation; NMOI, non-milk oral intake; NOWIA, non-oral water intake from the atmosphere; IAEA, the international atomic energy agency.

The estimated NMOI (mean and standard deviation) in a sample of 113 EBF Indonesian babies (6) under varying conditions and the corresponding 90th percentiles are presented in Table 2. The mean (standard deviation) calculated under IAEA assumptions was 50.9 (28.4) g/day, and the 90th percentile was 86.6 g/day. However, adjusting for variations in indoor temperature and atmospheric relative humidity in Table 2, the mean NMOI varied between 24.6 and 53.3 g/day, and the 90th percentile varied between 55.5 and 85.1 g/day. When different months in Yavatmal (13) were considered, the 90th percentile varied between 57.9 and 114.9 g/day (Supplementary Table S5). For the average annual temperature (26°C) and relative humidity (80%) in Indonesia, where the data were initially collected, the 90th percentile of the NMOI distribution was 62.5 g/day compared to 86.6 g/day when the standard assumptions were used.

**Table 2:** Distribution of non-milk oral intake (NMOI) calculated on data from 113 EBF children applying indoor temperature and relative humidity in different geographical locations (Ref: 11, 13).

Location	Indoor temperature (°C) Mean (SD)	Indoor relative humidity (%) Mean (SD)	Mean of NMOI (g/day)	SD of NMOI (g/day)	90th percentile value of NMOI (g/day)
Assumed value IAEA (Ref: 4)			50.9	28.4	86.6
Blacksburg (US)	22.3 (2.2)	42.2 (14.7)	53.3	25.6	85.1
Antananarivo (Madagascar)	23.5 (2.5)	57.3 (12.3)	29.3	25.3	60.5
Colombo (Sri Lanka)	29.4 (1.8)	73.3 (8.4)	50.0	25.5	81.8
Guatemala City (Guatemala)	22.8 (1.7)	61.6 (7.3)	46.6	25.4	78.1
Hong Kong (China)	24.0 (3.0)	63.7 (10.8)	43.7	25.3	75.3
Lima (Peru)	23.4 (3.1)	67.6 (8.4)	39.0	25.4	70.4
Singapore (Singapore)	27.0 (2.3)	67.6 (9.9)	43.3	25.4	74.8
Tuxtla Gutierrez (Mexico)	22.7 (2.4)	68.8 (7.2)	34.3	25.1	65.3
Delhi (India)	32.0 (1.4)	64.7 (16.6)	24.6	25.1	55.5
Dhaka (Bangladesh)	30.9 (1.4)	75.2 (7.9)	46.1	25.4	77.5
Faisalabad (Pakistan)	32.7 (3.5)	53.4 (12.4)	49.5	25.4	81.1
Yavatmal (India)	28.9 (4.6)	55.0 (19.6)	53.2	25.5	85.1

The distribution parameters were obtained from 113 EBF children.

SD, standard deviation; NMOI, non-milk oral intake; NOWIA, non-oral water intake from atmosphere; IAEA, international atomic energy agency; EBF, exclusively breastfed.

### 3.2. Variation in classification of non-EBF babies

Under standard IAEA assumptions (4) and using an NMOI > 86.6 g/day to identify non-EBF, 24.0% of two-month-old mixed-fed Indonesian babies (*n* = 221) (7) were classified as non-EBF. At an assumed temperature of 15°C, 38.5% and 19.0% were classified as non-EBF when relative humidity was set at 20% and 80%, respectively. When a temperature of 35°C was used, 32.1%, and 14.9% were classified as non-EBF when the relative humidity was set at 20% and 80%, respectively (Table 3).

**Table 3:** Change in proportion of non-EBF with different combinations of different temperatures and relative humidity (*n* = 221).

Temperature	Relative humidity	NOWIA (%)	% Non-EBF
Assumed value IAEA (Ref: 4)	Assumed value IAEA (Ref: 4)	0.063	24.0
15°C	20%	0.020	38.5
15°C	50%	0.049	25.8
15°C	80%	0.079	19.0
25°C	20%	0.023	37.1
25°C	50%	0.058	25.3
25°C	80%	0.092	17.2
35°C	20%	0.029	32.1
35°C	50%	0.072	20.4
35°C	80%	0.116	14.9

Children classified as non-EBF in a mixed-fed population of Indonesian children using NMOI > 86.6 g/day as the cut-off.

SD, standard deviation; NMOI, non-milk oral intake; NOWIA, non-oral water intake from the atmosphere; IAEA, international atomic energy agency; EBF, exclusively breastfed.

## 4. Discussion

This study examined the effects of atmospheric temperature and relative humidity on calculating NMOI using the DTM method. The assumed NOWIA increased with increasing temperature and relative humidity, resulting in an approximately 1 g/day decrease in the NMOI per unit increase in atmospheric temperature and relative humidity.

The DTM technique was initially developed to measure breast milk intake in babies; indeed, the accuracy of the computation of breast milk intake was intact and unaffected by temperature and relative humidity; this reinforces confidence in the technique used to quantify breast milk intake. However, several studies (14, 15) have been using NMOI to identify EBF in young children. A cut-off NMOI of 86.6 g/day for classification into EBF was published recently (7). The computed 1 g change of NMOI/day for one °C increase in average atmospheric temperature or one % relative humidity increase, constitutes a large methodological bias if ambient temperature and relative humidity variations are considered. Under restricted indoor conditions, the variation remained in the range of 50 g/day; this is a substantial variation given that the cut-off itself is 86.6 g/day.

Furthermore, it casts doubt on using cut-off-based deterministic identification of the EBF. The variation in proportion of children classified as EBF based on different temperatures and humidity demonstrates the possibility of misclassification if atmospheric temperature and humidity are not considered in the NMOI. A probability-based approach that assigns the probability of being EBF to every baby based on the distribution of NMOI in the standard EBF population would be better. However, adjustment for atmospheric temperature and relative humidity remains essential to obtain the correct distribution of the NMOI, even when using the probability-based approach. Liu et al. (7) clearly state that the proposed NMOI cut-off could change noticeably with temperature and relative humidity variations if the study were conducted in a different geography.

Atmospheric relative humidity had a more significant impact on the NMOI than temperature. When the relative humidity was increased by 60%, and the temperature was held constant, the prevalence of non-EBF, based on the previously defined NMOI cut-off of 86.6 g/day, was reduced by 19%. Ideally, the cut-off would be constant across geographical locations for the temperature-and relative humidity-adjusted NMOI.

To the best of our knowledge, this is the first study that closely examines, although theoretically, the implications of variations in atmospheric conditions on the computation of NMOI, which is becoming an increasingly important output of the DTM technique. However, a simple correction cannot be used to account for variations in temperature and relative humidity in the calculation of NMOI because of the complex calculations involved for NMOI. A web-based, user-friendly application could incorporate the actual atmospheric temperature and relative humidity, thus providing more accurate NMOI calculations. However, this would require a predictable association between meteorological data and indoor atmospheric conditions. This association needs to be understood to easily incorporate these adjustments in the NMOI calculations in the future. The calculation of NMOI can also be affected by the variation in atmospheric temperature and humidity that the baby is exposed to, during the DTM study period of 14 days, and this needs to be explored. To improve the accuracy of the estimation of NMOI, measurement of atmospheric conditions in the baby's immediate environment could be added to future protocols. However, the gain in accuracy must be balanced against the increased complexity of the method in the field as well as in the calculations. A limitation of the study is that the variation in NMOI presented in this paper is a theoretical demonstration and has not been validated in field setting.

In conclusion, this study demonstrates the need to consider atmospheric temperature and relative humidity when calculating NMOI.

## Data Availability

The original contributions presented in the study are included in the article/supplementary material, further inquiries can be directed to the corresponding author.
