# Supplementary material for: Effects of variations in atmospheric temperature and humidity on the estimation of exclusive breastfeeding status using the deuterium oxide dose-to-mother technique
Source: Front Pediatr. 2023 Nov 15;11:1188811. doi: 10.3389/fped.2023.1188811 (PMC10684944; doi:10.3389/fped.2023.1188811)
Supplement: Supplementary file 1 [file Table1.docx]

Supplementary Table 1. Characteristics of 9 children^*^ included in the demonstration of variations in non-milk oral intake (NMOI) and proportion of non-oral water intake from atmosphere (NOWIA) with varying temperature and relative humidity.

| Variable | Freq (%)/ Mean (SD) |
| --- | --- |
| Male sex | 1 (11%) |
| Age (days) | 112.6 (13.6) |
| Starting weight (kg) | 6.2 (0.8) |
| Milk intake (kg) | 1.49 (0.001) |

* Data taken from publicly available database of exclusively breast-fed children in Indonesia^6^

Supplementary Table 2. Characteristics of exclusively breastfed children (n=113) considered in the calculation of NMOI distributions and cutoffs

| Variable | Frequency (%)/ Mean (SD) |
| --- | --- |
| Male sex | 54 (47.8%) |
| Age (days) | 99.35 (23.18) |
| Starting weight (kg) | 6.10 (0.81) |
| Milk intake (kg) | 0.82 (0.16) |

* Data derived from the publicly available database of exclusively breast-fed infants in Indonesia^6^

Supplementary Table 3. Description of all infants (n=221) included in the calculation of percentage of non-EBF children

| Variable | Frequency (%)/ Mean (SD) |
| --- | --- |
| Male sex | 54 (47.8%) |
| Age (days) | 57.92 (10.31) |
| Starting weight (kg) | 4.88 (0.60) |
| Milk intake (kg) | 0.79 (0.38) |

* Data derived from the publicly available database of mixed infant population in Indonesia^6^

Supplementary Table 4. Variation in intake parameters by indoor temperature and relative humidity in different months in Yavatmal, India within a child

| Location | Indoor temperature (degree celsius)* | Indoor relative Humidity (%)* | NOWIA | Non-milk oral intake (g/day) |
| --- | --- | --- | --- | --- |
| Assumed value IAEA^(Ref:4)^ |  |  | 0.063 | 90.27 |
| January | 23.3 (3.3) | 49.5 (8.1) | 0.052 | 100.45 |
| February | 27.4 (3.4) | 39.0 (10) | 0.045 | 107.85 |
| March | 31.0 (4.1) | 29.0 (8.7) | 0.036 | 116.25 |
| April | 35.0 (3.6) | 21.7 (6.6) | 0.03 | 122.33 |
| May | 37.0 (3.1) | 32.1 (9.4) | 0.046 | 106.32 |
| June | 33.0 (2.9) | 60.1 (11.1) | 0.078 | 75.49 |
| July | 29.9 (2.4) | 72.8 (9.9) | 0.088 | 65.96 |
| August | 30.2 (2.2) | 72.0 (8.7) | 0.088 | 66.32 |
| September | 30.7 (2.6) | 73.9 (7.4) | 0.091 | 63.09 |
| October | 28.9 (2.5) | 65.2 (9.1) | 0.077 | 76.51 |
| November | 25.0 (3.5) | 54.5 (8.5) | 0.06 | 93.54 |
| December | 22.8 (3.3) | 47.0 (8.3) | 0.049 | 103.43 |

Water intake from milk (cl_mb_rs+ rm_rs) is a constant value 815 g/day, in the calculation

Water used in growth (rg_rs) is a constant value 9.28 g/day, in the calculation

NOWIA-proportion of non-oral water intake from atmosphere

Isotopic fractionation correction is fixed at 0.9919

*Values are mean (SD) of annual variation in indoor temperature and relative humidity^12^

Supplementary Table 5. Distribution of non-milk oral (NMOI) intake by indoor temperature and relative humidity in different months is Yavatmal, India

| Location | Indoor temperature (degree celsius) | Indoor relative humidity (%) | Mean of NMOI (g/day) | SD of NMOI (g/day) | 90^th^ percentile value of NMOI (g/day) |
| --- | --- | --- | --- | --- | --- |
| Assumed value IAEA^(Ref:4)^ |  |  | 50.9 | 28.4 | 86 |
| January | 23.3 (3.3) | 49.5 (8.1) | 61.3 | 26 | 94 |
| February | 27.4 (3.4) | 39.0 (10) | 68.1 | 26.1 | 100.9 |
| March | 31.0 (4.1) | 29.0 (8.7) | 75.8 | 26.3 | 108.9 |
| April | 35.0 (3.6) | 21.7 (6.6) | 81.6 | 26.5 | 114.9 |
| May | 37.0 (3.1) | 32.1 (9.4) | 66.7 | 26 | 99.1 |
| June | 33.0 (2.9) | 60.1 (11.1) | 38.1 | 25.5 | 69.6 |
| July | 29.9 (2.4) | 72.8 (9.9) | 29.2 | 25.3 | 60.5 |
| August | 30.2 (2.2) | 72.0 (8.7) | 29.6 | 25.4 | 61 |
| September | 30.7 (2.6) | 73.9 (7.4) | 26.6 | 25.3 | 57.9 |
| October | 28.9 (2.5) | 65.2 (9.1) | 39 | 25.5 | 70.7 |
| November | 25.0 (3.5) | 54.5 (8.5) | 54.7 | 25.9 | 87 |
| December | 22.8 (3.3) | 47.0 (8.3) | 64 | 26 | 96.6 |

The distribution parameters obtained from 113 EBF infants
