# Supplementary figures and images for: Effects of variations in atmospheric temperature and humidity on the estimation of exclusive breastfeeding status using the deuterium oxide dose-to-mother technique
Source: Front Pediatr. 2023 Nov 15;11:1188811. doi: 10.3389/fped.2023.1188811 (PMC10684944; doi:10.3389/fped.2023.1188811)

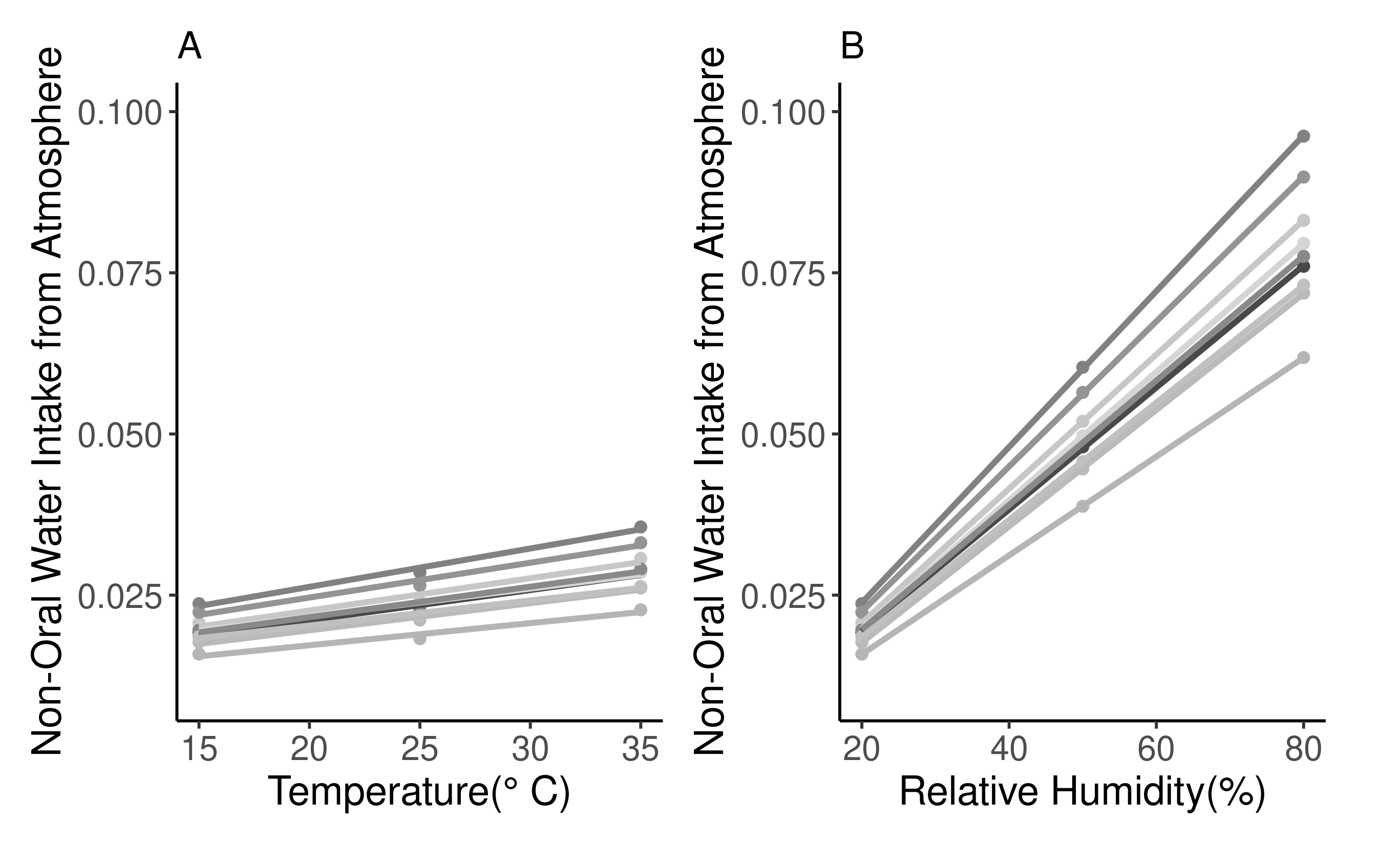

Supplement: Supplementary file 2 [file Image1.tiff]
